# Asymmetries in Gift-Exchange: The Role of Surprise When Wanting to Give/Receive Creative Gifts

**DOI:** 10.3390/bs16091522

**Published:** 2026-08-29

**Authors:** Rogelio Puente-Díaz, Judith Cavazos-Arroyo

**Affiliations:** 1 School of Business and Economics, Universidad Anáhuac México, Av. Universidad Anáhuac 46, Col. Lomas Anáhuac, Huixquilucan C.P. 52786, Mexico; 2 Centro Interdisciplinario de Posgrados, Universidad Popular Autónoma del Estado de Puebla, 21 Sur No. 1103, Col. Santiago, Puebla C.P. 72410, Mexico; cavazosjudith03@gmail.com

**Keywords:** asymmetries, gift-exchange, creativity, emotions, surprise

## Abstract

In the dynamic of gift-exchange, consumers play two important roles: givers and recipients. When they play both roles, the literature on gift-exchange has found asymmetries where what givers value is different from what gift recipients want. Across three experiments, we tested associations between the importance given to surprise and the desire for creative gifts and the generation of ideas for creative gifts. In experiment 1, participants were assigned to the role of the giver or recipient and rated how important it was for gifts to be original, effective (two dimensions of a creative gift), and surprising. Results showed that participants assigned to the role of the giver rated the originality and effectiveness of gifts as more important than participants assigned to the role of the recipient. This relationship was mediated by the importance placed on surprise, suggesting that givers placed more emphasis on surprising the recipient than recipients did. In addition to rating the importance of attributes for gift-exchange, participants were asked to generate three gift ideas that matched what they thought was important when giving or receiving gifts. Two independent judges rated the creativity of the gifts generated. Participants assigned to the role of the giver generated more creative ideas for gifts than participants assigned to the role of the recipient, providing behavioral evidence for the important role of creative gifts. Experiment 2 was an independent replication with a sample of consumers of a different socio-economic status and found additional support for the role of surprise and creative gifts in gift-exchange. In experiment 3, participants were assigned to recall either the best gift they had ever given or an ordinary gift. Participants assigned to the best-gift-ever-given condition recalled gifts that were more creative, as rated by two independent judges, than participants assigned to the ordinary-gift-given condition. In addition, results showed that giving more creative gifts made givers feel better about themselves in the form of moral self-perception. This relationship was mediated by the emotional reactions perceived by the recipient of the gift as inferred by the giver. The implications of the results are discussed.

## 1. Introduction

Gift-exchange is a highly popular consumption dynamic. Business scholars suggest that the size of the gift market is around USD 491.82 billion (Fortune Business Insights, 2026) and growing. Similarly, the creative process and creativity in general are popular characteristics in consumption (Ivcevic, 2025). We posit combining this conceptual and empirical literature to examine the concept of creative gifts. Creative gifts, by definition, should be original and useful. A website called Creative Gifts (2026) promises its customers help in finding them a unique, original gift for that special person. Another website, Konfetti (2026), ranks the top 10 creative gifts, offering options for that special one as well. Thus, it seems that creative gifts have an important place in gift-exchange and consumption. Besides the combination of two streams of research that, to our knowledge, have not been examined previously (see Givi et al., 2023 and Weinberger et al., 2025, for an empirical and conceptual overview), two related empirical questions drive our scientific enquiry: To what extent do gift givers and gift recipients equally value original and useful gifts? And what is the role of surprise in the exchange of creative gifts? To answer these two research questions and some related sub-questions, we conduct three experiments with young consumers from México. Hence, the purpose of the present investigation is threefold. First, we examine the role of creative gifts in general in the dynamics of gift-exchange. Second, we zoom in on the distinction between gift givers and recipients and examine the influence of this assigned role on the importance given to original and useful gifts, on eliciting surprise, and on the generation of creative gift ideas among young consumers from México. Third, we analyze the affective consequences of creative gifts for both givers and recipients. To achieve our research objective, we first explore the literature on gift-exchange with a special emphasis on the distinction between gift givers and recipients and what they want and value in gifts. Second, we explore the literature on creativity. Third, we combine the literature and posit research hypotheses before introducing our three experiments. Before beginning, an important caveat is in order. Our three experiments focus on young consumers from México. Thus, from now on, when we talk about consumers, we are limiting our explanations to young consumers from a collectivistic culture of honor such as México, who might have different cultural norms in gift-exchange. The examination of different cultural norms is beyond the scope of this article.

## 2. Gift-Exchange

The empirical literature on gift-exchange has grown considerably (Givi et al., 2023; Weinberger et al., 2025). As the field has grown, conceptual models have been developed to organize and propose directions for future research (Givi et al., 2023; Weinberger et al., 2025). In one of these models, Givi et al. (2023) identified five major themes that capture most of the empirical studies on gift-exchange: givers’ motivation, givers’ inputs, giver–recipient mismatches, value/creation reduction, and the great gift-giving context. To frame the current study, we focus on givers’ motivation and mismatches or asymmetries between givers and recipients in what they value when giving and receiving gifts.

Regarding motivation for gift-giving, the conceptual and empirical literature has identified several families of motives, including altruism, egoistic, social norms, and dyadic. We posit that when giving gifts, consumers want to surprise the gift recipient, a proxy of an altruistic motive and a self-presentation motive, especially if the recipient is psychologically close to the giver (Goodman & Lim, 2018). Surprise is a basic, epistemic emotion that comes from encountering unexpected information or stimuli (Widen, 2018). In the context of consumption, surprise is connected to consumer delight (Gupta & Gentry, 2018) that gives material objects and experiences meaning (Belk, 1996). The proposition that surprise is an important motivator is not new (Caplow, 1982, 1984). Indeed, Caplow (1984) suggested that surprise constitutes an unwritten rule in gift-exchange triggered by either showing more affection or knowledge than the gift recipient expects. In addition, the attention given to surprise in gift-exchange or self-gifting has increased in recent years (Gupta & Gentry, 2018; Gupta et al., 2020). What is new is the proposition that the desire to surprise in gift-exchange has manifestations in the attributes or characteristics of the gifts endorsed and in the generation of gift ideas for givers and recipients. The manifestations of the desire to surprise in gift-exchange could come in attributes considered important and in the generation of ideas for gifts to achieve the goal of surprising. Surprise can be experienced by anticipating a given consumption experience or by recalling a consumption experience. We focus on the anticipation of surprise and on the importance given to surprising rather than on the experience of the emotion itself.

Consistent with the mismatch between givers and recipients identified by Givi et al. (2023), we suggest another mismatch between givers and recipients. Specifically, we posit that the desire to surprise is not the same for gift givers as for gift recipients. Gift givers should place more emphasis on surprising gift recipients than the other way around because givers want to delight recipients more than recipients themselves would. The empirical literature shows indirect support for this proposition. For example, one study showed that gift givers focused mainly on the moment the gift is given rather than on the long-term consumption of the gift (Galak et al., 2016). While we could argue that individuals could consistently experience surprise when using a gift, surprise is more likely to occur when individuals receive the gift. Similarly, givers prefer gifts that can be enjoyed immediately rather than gifts that can be enjoyed in a more distant future (Yang & Urminsky, 2018). A gift that can be used immediately is more likely to generate surprise. In addition, there is empirical evidence that gift givers prefer to give gifts that have not been exchanged before, even though the gift recipients would be satisfied receiving a previously received gift (Givi, 2020; Givi & Galak, 2020). A never-before-given gift should elicit greater surprise and communicate deeper meaning than an already exchanged gift. Last, one study showed that gift givers placed more emphasis on the presentation of the gift rather than on the actual gift, and that gift recipients would have preferred the opposite (Yang & Urminsky, 2018). We posit that a well-presented gift is more likely to generate surprise than a gift that is not well-presented. Thus, there is enough empirical evidence to support our proposition that gift givers should place more emphasis on surprising the gift recipients than recipients would endorse, creating an intriguing asymmetry. To our knowledge, this constitutes a new asymmetry with important implications for creative gifts because finding and giving creative gifts might be a way to generate greater surprise and delight (Gupta & Gentry, 2018). In sum, the present study does not represent an advancement of gift-exchange theory. Instead, its value lies in identifying a new mismatch between gift givers and recipients; in using a relatively novel manifestation of this mismatch, the endorsement of the importance of giving original and useful gifts and the generation of gift ideas with such attributes; and in combining the literature on gift-exchange and creativity, which have not been integrated before. These potential contributions are in line with recent calls for future research (Givi et al., 2023). To explain these proposed connections, we now turn our attention to creativity and its implications for the dynamic of gift-exchange.

## 3. Creativity

Creativity is a multidisciplinary field of enquiry that has attracted the attention of educational (Beghetto, in press; Lebuda & Benedek, 2023), organizational (Reiter-Palmon et al., 2018), engineering (Cropley, 2015), and business scholars (Peppler, 2017), among others. Across the different disciplines, there is a consensus that creativity can be examined at the level of a product or service and that the product or service needs to have two characteristics to be creative: originality and usefulness (Stein, 1953). It is important to have both attributes to be considered creative (Simonton, 2012). An original product or service that does not solve a consumer’s problem is useless. Similarly, a useful product that lacks originality is also likely to lack differentiation to have a competitive advantage. Connecting this definition to the process of gift-exchange, we can examine whether and how gift givers and recipients value the attributes of originality and usefulness in gifts and how this endorsement manifests itself in the generation of ideas for gifts. Even though commercial enterprises offer creative gifts, the scientific literature has overlooked this integration. Givi et al. (2023) suggest that empirical studies have avoided using multiple perspectives in gift-exchange, probably because the field has evolved and matured slowly over time. Approaching gift-exchange from multiple perspectives requires familiarity with different sets of conceptual developments and empirical studies. This is why Givi et al. (2023) suggest approaching empirical studies from multiple perspectives as the field progresses. We see the integration of gift and creativity research as a relevant example of how empirical studies can integrate the separate literature.

In sum, we propose that, given that gift givers want to surprise recipients, they should show a stronger endorsement of the attributes of originality and usefulness in a gift than gift recipients. This is so because original gifts are probably more unexpected, and this unexpectedness should elicit surprise. Yet, originality alone might not be enough. Gifts also need to be useful because, in general, gift givers and recipients value utility (Yang & Urminsky, 2018). In addition, original gifts that are not useful would not be considered creative (Stein, 1953). Thus, we posit the following hypotheses:

**H1.**  *Individuals assigned to the role of the giver will show higher levels of endorsement of the attributes of originality and usefulness than individuals assigned to the role of the recipient*.

**H2.**  *The relationship proposed in H1 will be mediated by the importance given to surprise*.

The stronger endorsement of originality and usefulness by gift givers should lead to the generation of more creative ideas for gifts when thinking about future gifts. This is consistent with recent models of creative idea generation, suggesting that generating ideas involves spontaneous and controlled processes in which individuals try to achieve the goal of generating ideas that are both original and useful (Benedek et al., 2023). Thus, gift givers should generate more creative ideas for gifts after endorsing the attributes of originality and usefulness. This proposition seeks to show that the importance given to originality and usefulness goes beyond endorsement and has manifestations in idea generation for creative gifts. We propose that:

**H3.**  *Consistent with H1 and H2, individuals assigned to the role of the giver will generate more creative ideas for gifts as rated by independent judges than individuals assigned to the role of the recipient when thinking about the hypothetical scenario of gift-exchange with a special person*.

As our conceptual developments have elaborated, we suggest greater emphasis on creative gifts by givers than recipients. Two interesting and relevant questions emerge: What are the emotional advantages of receiving creative gifts for gift recipients beyond the initial elicitation of surprise? And what are the emotional advantages of giving creative gifts for gift givers? Empirical research has shown that gifts have emotional consequences (Puente-Díaz & Cavazos-Arroyo, 2021). We posit that gift givers would have an emotional advantage in giving creative gifts by perceiving the emotions elicited in the gift recipients. This proposition comes from conceptual developments on surprise and gift-exchange, which suggest that eliciting surprise could lead to a positive emotional peak for both givers and recipients (Gupta & Gentry, 2018). In addition, the justification for this connection is that, given that givers emphasize surprising recipients, they should focus their attention on the emotional reactions of the recipients to experience their emotional peak (Gupta & Gentry, 2018). That is, it feels good to see the gift recipient experiencing positive emotions from the gift. Thus, we focus on assessing the emotions in gift recipients inferred by the gift giver and not actually experienced by gift recipients. We posit that noticing positive emotions in gift recipients should be associated with givers feeling good about themselves. This positive feeling about the self could be manifested in high moral self-perception. To test this, we use recalled gifts and posit two hypotheses:

**H4.**  *When individuals are assigned to recall the best gift ever given, they will recall gifts with greater creativity than individuals assigned to recall an ordinary gift.*

**H5.**  *Creative gifts, as rated by two independent judges, will have an indirect positive relationship with moral self-perceptions experienced by the giver by having a direct positive relationship with the emotions experienced by gift recipients through the perceptions of the givers.*

In sum, the purpose of the present investigation is threefold. First, we examine the role of creative gifts in the dynamics of gift-exchange. Second, we zoom in on the distinction between gift givers and recipients and examine the influence of this assigned role on the importance given to original and useful gifts, on eliciting surprise, and on the generation of creative gift ideas. Third, we analyze the affective consequences of creative gifts for both givers in the form of moral self-perception and recipients. To achieve our objectives and test our hypotheses, three experiments are conducted. We explain these experiments next.

## 4. Overview of Studies

We had the goal of conducting three experiments. We used G*Power 3.1.9.7 (Faul et al., 2007) to estimate the sample size needed with the following characteristics: effect size |*p*| = 0.30, α error probability = 0.05 (two-tailed), power = 0.95, and two groups. The results showed a sample size of 148 participants. We used 148 as a lower bound, seeking to have a larger sample size because in experiments 1 and 2, in addition to group differences, we also conducted a mediation analysis. Experiment one focused on testing hypotheses one, two, and three. Experiment two was a conceptual replication with participants of a lower socio-economic status. Experiment three complemented the results of the first two experiments by testing hypotheses four and five. In all experiments, we used samples of convenience. Even though these samples had young consumers who were our target of interest, it is an important limitation worth keeping in mind when interpreting the results. All experiments were approved by the human subjects committees of our universities (2025-Ana-165, 2025-UPAEP-118 and 2025-Ana-196).

## 5. Method of Study 1

### 5.1. Participants

Participants were 196 consumers (99% in the 18–25 age group, 72% female) from a private school in México. Regarding socio-economic status, 17% reported belonging to a high status, 60% to a high/medium status, and 19% to a medium status (the remaining 4% reported a lower level or did not want to report it). Participants voluntarily agreed to participate in this study without any financial compensation and indicated their consent by clicking on the relevant link sent to them.

### 5.2. Measures and Procedures

Participants followed this sequence of instructions, tasks, and measures to be completed, trying to create a realistic scenario to report their endorsement of gift attributes and generate ideas for gifts.

Step 1: Participants first read the following text and were asked to write down the name of a special person to give or receive a gift: “Gifts are a way to give or receive love and care from and to someone special. We would like you, please, to think of this special person and write her or his name down”.

Step 2: After reporting the name of their special person, participants were randomly assigned to one of the following two conditions:

Gift-giver condition: The instructions were: “Gifts can have different characteristics. Let us suppose that you want to buy a gift for this special person you just mentioned for around 1000 pesos (around $55 American dollars). Thinking of this special person, how important are the following characteristics to you when buying and giving a gift to this special person? The gift is for this special person”. Afterward, participants rated the following attributes presented randomly on a scale from 1 (not important at all) to 10 (very important): originality, usefulness, and surprise. 

Gift-recipient condition: The instructions were: “Gifts can have different characteristics. Let us suppose that the special person you just mentioned wants to buy you a gift for around 1000 pesos (around $55 American dollars). Thinking of this special person, how important are the following characteristics to you when buying and receiving a gift from this special person? The gift is for you”. Afterward, participants rated the same three attributes as mentioned above. Thus, we had three indicators for each participant. The only difference between the two experimental conditions was the assigned role: giver or recipient.

Step 3: Participants in the gift-giving condition read the following text: “Now that you have shared what is important when buying a gift for this special person, we would like to think of three gift ideas with such characteristics. What would you like to buy and give to this special person? My first idea is:”.

Participants in the gift-recipient condition read the following text: “Now that you have shared what is important when this special person buys a gift for you, we would like to think of three gift ideas with such characteristics. What would you like this special person to buy and give to you? My first idea is:”.

Thus, in both scenarios, participants generated three gift ideas that have the characteristics they just rated. The only difference was that in one condition, the gifts were for a special person and in the other condition, the gifts were from this special person. Thus, we had three gift ideas for each person. The literature on creativity often uses judges to evaluate the creativity of ideas (Kaufman, 2009). We decided to follow this procedure. Specifically, two judges with more than 15 years of experience in marketing and consumption and blind to the research hypotheses and objectives of the experiment read all gift ideas and evaluated them on a scale from 1 (not original/not useful at all) to 10 (very original/very useful). Thus, we had two scores for each gift idea. To assess the consistency of the scores generated by both raters, we calculated the intraclass correlations focusing on consistency agreement. Results showed acceptable values, 0.77 (CI = 0.74, 80) and 0.82 (CI = 0.77, 0.86), for originality and usefulness.

Step 4: Last, participants completed a short demographic questionnaire reporting their age group, sex, and socio-economic status. The entire sequence of tasks and measures took about 15 min to complete.

### 5.3. Results

We first analyzed the ratings of originality and usefulness as a function of the experimental condition: giver versus recipient. Given that a creative gift must be original and useful, we multiplied the scores of both variables to obtain an indicator of importance of creativity, as has been done in previous studies in creativity research (Puente-Díaz et al., 2026) and documented in conceptual developments (Simonton, 2012). The rationale for multiplying both scores is that a gift needs to have both characteristics to be creative. If it does not, the product of multiplying these two variables would be zero (see Simonton, 2012 for an explanation). We had some missing values in some variables, thus the degrees of freedom reported in the analyses might be different from the total sample size reported.

We conducted a one-way Analysis of Variance (ANOVA) with the experimental condition with two levels as the independent variable and the newly created variable indicating the importance of creative gifts as the dependent variable. Results showed that participants assigned to the role of the giver reported greater levels of endorsement of creative gifts than participants assigned to the role of the recipient, F(1, 195) = 27.18, *p* < 0.001, Cohen D’s = 0.74. Mgiver = 77.36, SD = 19.99 vs. Mreci = 58.80, SD =29.09. To test the mediation hypothesis, we coded the gift-giver condition as 1 and the gift-recipient condition as 0. We followed the guidelines set by Hayes (2022, model 4). Results first showed a significant association between the experimental condition and surprise (b = 0.99, *p* < 0.001). Results then showed a positive association between surprise and endorsement of creative attributes while controlling for the association with the experimental condition (b = 5.63, *p* < 0.001). The bootstrapped confidence (5000 iterations) interval for the indirect association did not contain zero, 5.58, SE= 1.81 |2.23, 9.41|. The significance of the mediation model indicates that participants assigned to the role of the giver put more emphasis on creative attributes in gifts than individuals assigned to the role of the recipient because they assigned greater importance to the emotion of surprise.

We then focused on analyzing the creativity of the gift ideas generated by each participant as a function of their experimental condition. To obtain a creativity score, we multiplied the original score by the usefulness score generated by the two judges and computed the mean of the three gift ideas (Puente-Díaz et al., 2026). We conducted a one-way Analysis of Variance (ANOVA) with the experimental condition with two levels as the independent variable and creativity scores from the gift ideas generated by each participant as the dependent variable. Results showed that participants assigned to the role of the giver generated ideas with greater creativity than participants assigned to the role of the recipient, F (1, 195) = 14.61, *p* < 0.001, Cohen’s d = 0.54. Mgiver = 40.22, SD = 11.59 vs. Mreci = 34.77, SD =8.00. These results suggest that givers generated ideas for creative gifts consistent with the assigned importance of giving gifts that are both original and useful.

### 5.4. Brief Discussion

Our results showed that participants randomly assigned to the role of the giver rated the importance of giving creative gifts as higher than participants assigned to the role of the recipient. This was because givers placed more importance on surprising the gift recipient. In addition, participants assigned to the role of the giver generated more creative ideas for gifts than participants in the recipient condition. These results supported hypotheses 1, 2, and 3. Given that our results showed interesting, intriguing findings, we saw the need for independent replication. In addition, to enhance the validity of our results, we conducted this replication with consumers of a lower socio-economic status.

## 6. Method of Study 2

### 6.1. Participants

Participants were 281 consumers (78% in the 18–25 age group, 16% in the 26–35 age group, 72% female) from a private school in México. Regarding socio-economic status, 4% reported belonging to a high status, 25% to a high/medium status, 49% to a medium status, and 20% to a medium/low status (the remaining 2% reported a lower level or did not want to report it). Thus, this sample belonged to a greater extent to a middle socio-economic status than consumers from study 1. Participants voluntarily agreed to participate in this study without any financial compensation and indicated their consent by clicking on the relevant link sent to them.

### 6.2. Measures and Procedures

The measures and procedures were identical to study 1. Two independent raters evaluated all the gift ideas generated by participants with acceptable levels of consistency (intraclass correlation of 0.79 and 0.80 for originality and usefulness, respectively).

### 6.3. Results of Study 2

**Figure 1:**
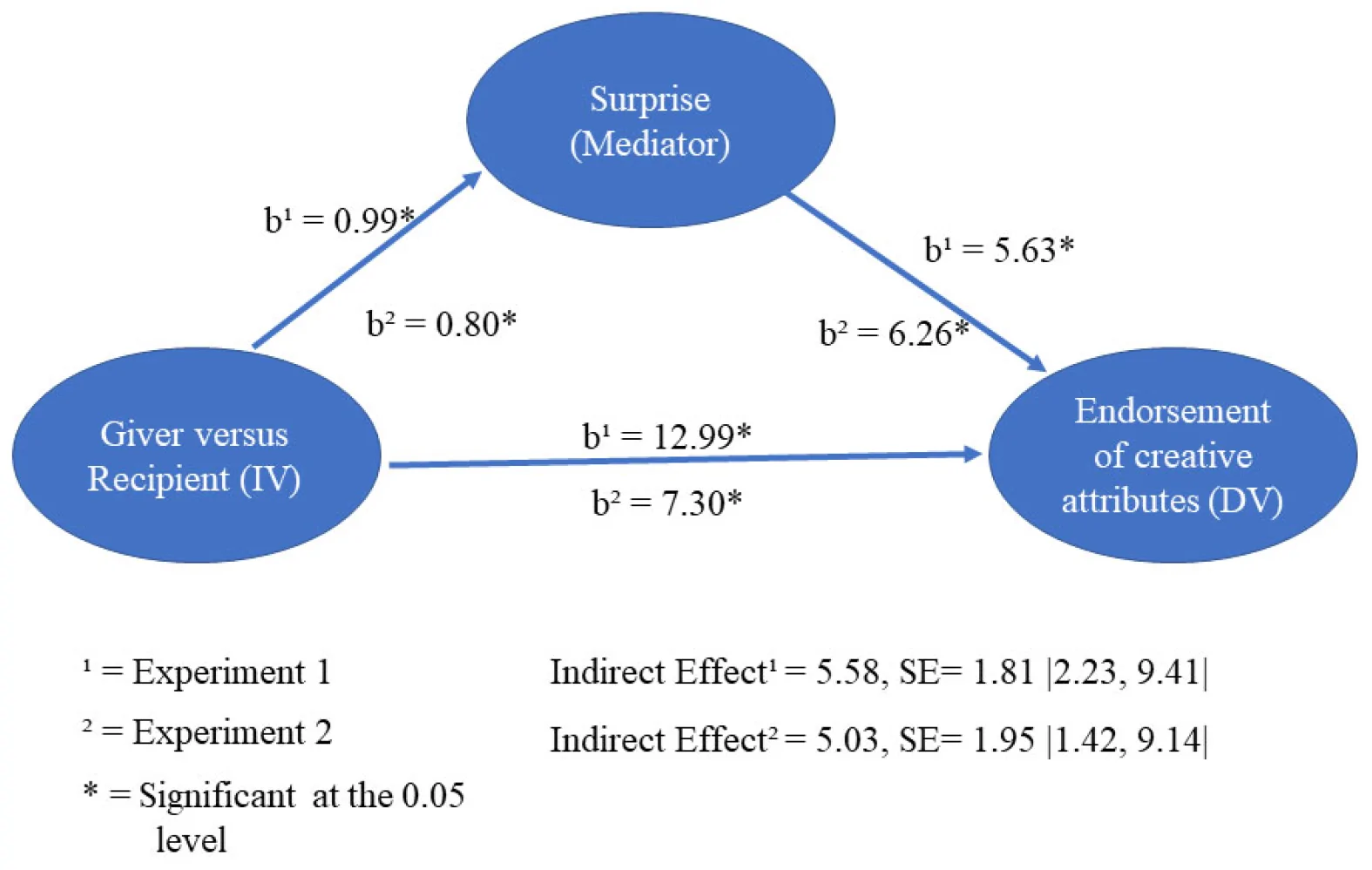
Summary of results of mediation model of studies 1 and 2.

As in study 1, we first analyzed the ratings of originality and usefulness as a function of the experimental condition: giver versus recipient. We multiplied the scores of originality and usefulness to obtain an indicator of the importance of creativity in gifts, as in study 1 and previous studies (Puente-Díaz et al., 2026)^1^. We had some missing values in some variables; thus, the degrees of freedom reported in the analyses might be different from the total sample size reported. We conducted a one-way Analysis of Variance (ANOVA) with the experimental condition with two levels as the independent variable and the newly created variable indicating the importance of creative gifts as the dependent variable. Results showed that participants assigned to the role of the giver reported greater levels of importance of creative gifts than participants assigned to the role of the recipient, F(1, 281) = 12.43, *p* < 0.001, Cohen’s d = 0.43. Mgiver = 62.90, SD = 26.91 vs. Mreci = 50.57, SD =30.16. To test the mediation hypothesis, we followed the same procedures and guidelines as in study 1 (Hayes, 2022, model 4). Results first showed a significant association between the experimental condition and surprise (b = 0.80, *p* < 0.001). Results then showed a positive association between surprise and endorsement of creative attributes while controlling for the association with the experimental condition (b = 6.26, *p* < 0.001). The bootstrapped confidence interval for the indirect association did not contain zero, 5.03, SE= 1.95 |1.42, 9.14|. The significance of the mediation model indicates that participants assigned to the role of the giver placed more emphasis on surprise, which was associated with placing more importance on gifts that are original and useful (see Figure 1 for a summary of results).

We then focused on analyzing the creativity of the gifts generated by each participant as a function of their experimental condition, as in study 1. We multiplied the original score by the usefulness score and computed the mean of the three gift ideas (Puente-Díaz et al., 2026). We conducted a one-way Analysis of Variance (ANOVA) with the experimental condition with two levels as the independent variable and creativity scores coming from the gift ideas generated by each participant as the dependent variable. Results showed that participants assigned to the role of the giver generated ideas with greater creativity than participants assigned to the role of the recipient, F (1, 279) = 8.04, *p* = 0.005, Cohen’s d = 0.34. Mgiver = 33.74, SD = 8.76 vs. Mreci = 31.04, SD =7.18. What these results suggested was that givers generated ideas with greater creativity than recipients, consistent with the reported importance of giving gifts that are both original and useful.

### 6.4. Brief Discussion

Our results replicated the findings from study 1, increasing the validity of our results by providing additional support for hypotheses 1, 2, and 3. When assigned to the role of the gift giver, participants reported that it was more important to give creative gifts and generated more creative ideas for gifts than participants assigned to the role of the gift recipient. Yet, it is worth mentioning that the effect sizes were smaller than in study 1. The first two experiments involved generating ideas for future creative gifts. To complement our results, we focused on the past and asked participants to either recall the best gift they had ever given or an ordinary gift and examined the influence of this experimental manipulation on the actual creativity of the gifts recalled. The idea of assessing the best gift or the perfect gift comes from the seminal work of Belk (1996). Yet, the best gift only partially captures the seminal conceptual work of Belk (1996). This third study also gave the chance to examine creative gifts in realistic as opposed to hypothetical scenarios. In addition, the emotional implications for the gift giver and recipient were examined.

## 7. Method of Study 3

### 7.1. Participants

Participants were 278 consumers (62% in the 18–25 age group, 25% in the 26–35 age group, 13% in the 36–50 age group, 55% female) from two private schools in México. Participants voluntarily agreed to participate in this study without any financial compensation and indicated their consent by clicking on the relevant link sent to them.

### 7.2. Measures and Procedures

Participants followed this sequence of instructions, tasks, and measures to be completed.

Step 1: Participants were randomly assigned to one of the two following experimental conditions.

Best-gift-condition: Participants read the following text: “We all have given a gift in our lives. We would like you to please think about the best gift you have ever given and describe it in the space below.”

Ordinary-gift-condition: Participants read the following text: “We all have given a gift in our lives. We would like you to please think about a common, ordinary gift you have given and describe it in the space below.”

All gifts recalled in both conditions were scored on originality and usefulness by two judges with more than 15 years of experience in marketing and consumption using the same scale as studies 1 and 2. The intraclass correlation showed acceptable levels of consistency, 0.80 and 0.82.

Step 2: Participants read the following instructions. “The following questions are about how you think the person receiving the gifts felt”. The questions were: at the moment of receiving the gift, how grateful/surprised/glad do you think the person receiving the gift felt? All three questions were reported on a scale from 1 (not at all) to 10 (very). All three questions were averaged (α = 0.84) to obtain a mean of positive emotions felt by the gift recipient. We posit that asking the gift giver about the emotions experienced by the recipient is a valid way of assessing the emotions experienced by the recipient because, when giving gifts, the emotions experienced by recipients are important. Yet, it is important to keep in mind that we are not assessing the actual emotions experienced by the gift recipient.

Step 3: Participants read the following text. “Now we are going to talk about how you felt when giving the best gift/an ordinary gift. We are talking now about your feelings.” The questions were: I felt like a worthy person/grateful for others/considerate person/good person on a scale from 1 (I did not feel like that at all) to 10 (I completely felt like that). All four questions were averaged (α = 0.88) to obtain a mean of moral self-perception experienced by the gift giver (Vaz et al., 2026).

Step 4: Participants completed a brief questionnaire reporting their age group and sex.

### 7.3. Results

We first calculated a creativity score for the gifts recalled by multiplying the originality scores from the judges by the usefulness scores. This variable served as our dependent variable, and the experimental condition with two levels as our independent variable. Results of a one-way Analysis of Variance (ANOVA) showed that participants assigned to the condition of best-gift recalled more creative gifts than participants assigned to the ordinary-gift condition, F(1, 266) = 111.96, *p* < 0.001, Cohen’s d = 1.04, Mbest = 33.37, SD = 16.66 vs. Mordi = 19.97, SD = 7.23. We then tested a mediation model hypothesizing that the creativity scores of the gifts had a positive, indirect relationship with moral self-perceptions experienced by the gift giver, through its positive relationship with positive emotions in the gift recipient inferred by the gift giver. Results showed a significant yet small indirect effect, 0.0084, SE = 0.0023 |0.004, 0.013| (see Table 1 for descriptive statistics of all experiments). Thus, in terms of affective benefits, we found that both gift givers and recipients experienced higher positive emotions when recalling having given creative gifts.

### 7.4. Brief Discussion

Participants assigned to the condition of best-gift-given recalled more creative gifts than participants assigned to recall an ordinary gift, supporting hypothesis 4. The creativity of the gift was positively and indirectly related to moral self-perceptions experienced by the gift giver through its positive, direct relationship with positive emotions experienced by the gift recipient, inferred by gift givers, supporting hypothesis 5. However, the size of the mediation effect was small, suggesting that conclusions should be drawn cautiously.

## 8. General Discussion

The purpose of the present investigation was threefold. First, we examined the role of creative gifts in the dynamics of gift-exchange. Second, we zoomed in on the distinction between gift givers and recipients and examined the influence of this assigned role on the importance given to original and useful gifts, on eliciting surprise, and on the generation of creative gift ideas. Third, we analyzed the affective consequences of creative gifts for both givers and recipients. Five hypotheses were tested and supported in three experiments. The theoretical and applied implications were discussed.

### 8.1. Theoretical Implications

As stated in our introduction, gift-exchange is a popular consumption dynamic around the world. Similarly, creativity is a highly valued outcome in business. Combining both streams of research, we posited an asymmetry in the importance given to original and useful gifts between givers and receivers. Our results showed that gift givers endorsed more strongly the attributes of originality and usefulness than gift recipients did, supporting hypothesis one. These findings made a small contribution to the growing literature on asymmetries between givers and recipients identified in a recent literature review (see Givi et al., 2023 for a conceptual overview of the empirical literature; see Qiu & Lu, 2025 for a recent empirical article). In addition, we posited that the higher endorsement of the attributes of originality and usefulness was in part driven by wanting to surprise the gift recipients. Our results showed that surprise mediated the relationship between the assigned role, giver or recipient, and the endorsement of originality and usefulness, supporting hypothesis two. These findings were consistent with conceptual developments on surprise in romantic relationships (Gupta & Gentry, 2018) and in self-gifting (Gupta et al., 2020), suggesting that surprise plays a prominent role in gift-exchange. In addition, consumers assigned to the role of the giver generated gifts with greater creativity than participants assigned to the role of the recipient, supporting hypothesis three. These findings allowed us to observe that the greater emphasis on creative gifts shown by gift givers went beyond stated preferences and had a behavioral manifestation in the form of creative ideas. These results were consistent with the proposition that idea generation is driven by the goal of generating ideas that are original and useful, in which spontaneous and controlled cognitive processes interact (Benedek et al., 2023). It is worth noting that the effect size for the endorsement of creative gifts was larger than the effect size of the actual generation of creative ideas for gifts. These results suggested that it was easier to think about attributes than to generate gift ideas with those characteristics. The problem space of “creative gifts” might be limited in consumers’ minds. This might be why commercial enterprises offer help to consumers in finding a creative gift.

The above findings were replicated with a sample of consumers of a lower socio-economic status, lending further support for our conceptual developments. Yet, it is worth noting that compared to findings from experiment 1, the effect sizes were smaller. Our intention was not to explore differences between socio-economic statuses, but to further validate our results. Yet, future research might want to explore differences in gift-exchange as a function of socio-economic status (Fiske & Markus, 2012). Qualitative examination of the gift ideas from experiment 2 showed less elaborate ideas than in experiment 1.

In addition, our results showed that when recalling gifts, consumers assigned to bring to mind the best gift ever given recalled more creative gifts than consumers assigned to bring to mind an ordinary gift, supporting hypothesis four. It was important to conduct experiment three because the first two experiments focused on hypothetical scenarios for future gifts. Instead, experiment three focused on recalled gifts and found incremental evidence. In addition, consumers who recalled their best gifts ever given showed higher levels of moral self-perception, explained by the higher levels of positive emotions experienced by the receiver, supporting hypothesis five. Our results were consistent with conceptual developments on surprise and its connection with delight and how this delight often leads to an emotional peak for both the gift giver and the recipient (Gupta & Gentry, 2018).

### 8.2. Applied Implications

We propose two applied implications. First, even though our findings support the importance of creative gifts, these types of gifts might not be for everyone. The empirical literature shows a great deal of individual differences among consumers and a great deal of variability as a function of the occasion for gifts (Givi et al., 2023). Given this, a creative gift might not be for everyone across all gift opportunities. Thus, brands should target creative gifts to individuals likely to value these types of gifts such as individuals high in openness to experience. Second, brands of products and services might be well-advised to give consumers options to surprise someone special. One way to achieve this is by carefully choosing and promoting gifts that are original and useful to consumers. In addition, brands can promote the emotional benefits of giving creative gifts by showing images of consumers feeling like a worthy person for giving creative gifts.

### 8.3. Limitations

Our experiments had several limitations. First, most of our consumers were young, coming from two private schools in México. This demographic does not represent the entire population of México well. Thus, future studies should use samples from other demographics, older consumers and consumers of a low socio-economic status to test how important the role of creative gifts is. Second, experiments 1 and 2 used hypothetical scenarios. These types of scenarios are often used in consumer research, yet future studies should focus on testing more realistic scenarios. Future studies could recruit consumers who are about to buy a gift and randomly assign them to buy a more creative versus a non-creative gift and examine the benefits of being assigned to give a creative gift. Third, one major strength of this study was the use of experiments to manipulate types of roles and types of gifts, givers versus recipients, and best gift versus ordinary gift, yet we were not able to capture the dynamic of gift-exchange longitudinally. Future studies could combine experiments with diary methodologies to examine how the selection of creative gifts and the emotional benefits of creative gifts unfold over time. Fourth, in experiment three, we did not directly assess the emotions experienced by the gift recipient. Instead, we relied on the inferences made by the gift giver. Future studies could use dyadic designs to assess the emotions experienced by gift recipients when receiving gifts from givers. Fifth, in experiment 3, the best-gift condition could be confounded with the memorability of more creative gifts. Thus, we cannot establish what elicited the emotional advantages in either givers or recipients as inferred by givers: remembering the best gift ever given or the memory advantage of more creative gifts. Last, we only used two judges to rate all ideas for creative gifts. Even though we do not anticipate major changes in the results from adding judges, future studies should try to secure more judges to ensure reliable estimates of the creativity of the ideas generated.

In sum, across three experiments asking consumers to think about future gifts and past gifts, we found strong evidence for the role of creative gifts. Specifically, gift givers placed more emphasis on giving creative gifts than recipients. This emphasis manifested in more creative ideas for gifts. In addition, recalling more creative gifts had emotional benefits for the giver, in the form of higher moral self-perception, and the recipient. As shown in different literature reviews and conceptual developments (Branco-Illodo et al., 2025; Givi et al., 2023; Weinberger et al., 2025), research on gift-exchange is growing and will continue to expand because it represents a relevant research theme and because it has many applied implications for businesses.

## Figures and Tables

**Table 1:** Descriptive statistics for experiments 1, 2, and 3.

**Experiment 1**	**Giver**				**Recipient**	
	**Mean**	**SD**	**95% CI**	**Mean**	**SD**	**95% CI**
Endorsement of creative attributes	77.36	19.99	73.38, 81.35	58.8	29.09	52.94, 64.67
Surprise	8.61	1.46	8.32, 8.90	7.62	2.41	7.13, 8.10
Creativity scores	40.22	11.59	37.91, 42.53	34.77	8.00	33.16, 36.39
**Experiment 2**	**Giver**			**Recipient**		
Endorsement of creative attributes	62.90	26.91	57.95, 67.84	50.57	30.16	45.93, 55.21
Surprise	7.68	2.23	7.27, 8.09	6.88	2.61	6.48, 7.28
Creativity scores	33.74	8.76	32.13, 35.35	31.04	7.18	29.93, 32.14
**Experiment 3**	**Best gift**			**Ordinary gift**		
Creativity scores	33.37	16.66	34.67, 40.07	19.97	7.23	18.66, 21.29

## Data Availability

All data sets are available upon request from the first author.

## Note

1 One reviewer rightfully recommended the use of Multilevel Modeling to analyze the results because creative ideas were nested within observation. The results do not change the conclusions but they are reported in Appendix A at the end of the article.

## Appendix A

Given that participants generated three gift ideas, the ideas were nested within observations or time. Thus, as a robust check, we conducted a multilevel model with the creativity of the gift ideas generated as the dependent variable, with three observations and the experimental condition as the independent variable. We followed the guidelines set by Geiser (2013). We used the two-level random analysis command in Mplus 7.11 with a Robust Maximum Likelihood (MLR) estimation. This model is also known as the random intercept and slope model (See Geiser, 2013). Results from experiment 1 showed a positive association between the creativity in the gift ideas generated, as rated by judges, and the experimental condition (b = 7.72, *p* = 0.005). These results showed that participants in the gift-giver condition generated more creative gift ideas than participants in the gift-recipient condition.

We followed the same strategy for experiment 2. Results from experiment 1 showed a positive association between the creativity in the gift ideas generated, as rated by judges, and the experimental condition (b = 4.46, *p* = 0.017). These results showed that participants in the gift-giver condition generated more creative gift ideas than participants in the gift-recipient condition, as in study 2. All data are available upon request from the first author.

## References

[B1-behavsci-16-01522] Beghetto R. A., Beghetto R. A. (in press). Human creativity and GenAI in education: A conceptual framework. Oxford handbook of human creativity and generative artificial intelligence in education.

[B2-behavsci-16-01522] Belk R. W., Otnes C., Beltramini R. F. (1996). The perfect gift. Gift giving: A research anthology.

[B3-behavsci-16-01522] Benedek M., Beaty R. E., Schacter D. L., Kenett Y. (2023). The role of memory in creative ideation. Nature Reviews Psychology.

[B4-behavsci-16-01522] Branco-Illodo I., Heath T., Otnes C., Givi J. (2025). Defining and delineating mindful gifting: Review and research agenda. Psychology & Marketing.

[B5-behavsci-16-01522] Caplow T. (1982). Christmas gifts and kin networks. American Sociological Review.

[B6-behavsci-16-01522] Caplow T. (1984). Rule enforcement without visible means: Christmas gift giving in Middletown. American Journal of Sociology.

[B7-behavsci-16-01522] Creative Gifts (2026). Our passion for unique gifts.

[B8-behavsci-16-01522] Cropley D. H. (2015). Creativity in engineering: Novel solutions to complex problems.

[B9-behavsci-16-01522] Faul F., Erdfelder E., Lang A.-G., Buchner A. (2007). G*Power 3: A flexible statistical power analysis program for the social, behavioral, and biomedical sciences. Behavior Research Methods.

[B10-behavsci-16-01522] Fiske S., Markus H. R. (2012). Facing social class: How societal rank influences interaction.

[B11-behavsci-16-01522] Fortune Business Insights (2026). Gift retailing market.

[B12-behavsci-16-01522] Galak J., Givi J., Williams E. F. (2016). Why certain gifts are great to give but not to get: A framework for understanding errors in gift giving. Current Directions in Psychological Science.

[B13-behavsci-16-01522] Geiser C. (2013). Data analysis with Mplus.

[B14-behavsci-16-01522] Givi J. (2020). (Not) giving the same old song and dance: Givers’ misguided concerns about thoughtfulness and boringness keep them from repeating gifts. Journal of Business Research.

[B15-behavsci-16-01522] Givi J., Birg L., Lowrey T. M., Galak J. (2023). An integrative review of gift-giving research in consumer behavior and marketing. Journal of Consumer Psychology.

[B16-behavsci-16-01522] Givi J., Galak J. (2020). Selfish prosocial behavior: Gift-giving to feel unique. Journal of the Association for Consumer Research.

[B17-behavsci-16-01522] Goodman J. K., Lim S. (2018). When consumers prefer to give material gifts instead of experiences: The role of social distance. Journal of Consumer Research.

[B18-behavsci-16-01522] Gupta A., Eilert M., Gentry J. W. (2020). Can I surprise myself? A conceptual framework of surprise self-gifting among consumers. Journal of Retailing and Consumer Services.

[B19-behavsci-16-01522] Gupta A., Gentry J. W., Minowa Y., Belk R. (2018). If you love me, surprise me. Gifts, romance, and consumer culture.

[B20-behavsci-16-01522] Hayes A. F. (2022). Introduction to mediation, moderation, and conditional process analysis.

[B21-behavsci-16-01522] Ivcevic Z. (2025). The creative choice: The science of making decisions to turn ideas into action.

[B22-behavsci-16-01522] Kaufman J. (2009). Creativity 101.

[B23-behavsci-16-01522] Konfetti (2026). The top 10 gifts- for people who love something special.

[B24-behavsci-16-01522] Lebuda I., Benedek M. (2023). A systematic framework of creative metacognition. Physics of Life Reviews.

[B25-behavsci-16-01522] Peppler K., Kaufman J. C., Glaveneau V. P., Baer J. (2017). Creativity in business and technology. The cambridge handbook of creativity across domains.

[B26-behavsci-16-01522] Puente-Díaz R., Cavazos-Arroyo J. (2021). Experiential gifts as meaningful moments and memories: Their influence on nostalgia and relive intention. Psychology & Marketing.

[B27-behavsci-16-01522] Puente-Díaz R., Cavazos-Arroyo J., Puerta-Sierra L. (2026). Expectation and perceived performance during the creative ideation process: The interplay between cognitive, metacognitive, and motivational components. Thinking Skills and Creativity.

[B28-behavsci-16-01522] Qiu T., Lu J. (2025). Gift givers over avoid sending used gifts. International Journal of Consumer Studies.

[B29-behavsci-16-01522] Reiter-Palmon R., Kennel V. L., Kaufman J. C. (2018). Individual creativity in the workplace.

[B30-behavsci-16-01522] Simonton D. K. (2012). Taking the U.S. Patent Office criteria seriously: A quantitative three-criterion creativity definition and its implications. Creativity Research Journal.

[B31-behavsci-16-01522] Stein M. I. (1953). Creativity and culture. The Journal of Psychology: Interdisciplinary and Applied.

[B32-behavsci-16-01522] Vaz A., Mata A., Critcher C. R. (2026). Absolute moral perceptions of the self and others: People are bad, a person is good, I am great. Journal of Personality and Social Psychology.

[B33-behavsci-16-01522] Weinberger M. F., Baskin E., Gunasti K. (2025). Relational gifting: Conceptual frameworks and an agenda for a new generation of research. Journal of Consumer Research.

[B34-behavsci-16-01522] Widen S. C., Barret L. F., Lewis M., Haviland-Jones J. M. (2018). The development of children’s concepts of emotion. The handbook of emotions.

[B35-behavsci-16-01522] Yang A. X., Urminsky O. (2018). The smile-seeking hypothesis: How immediate affective reactions motivate and reward gift giving. Psychological Science.

